# Effects of sitting balance training with a wedge on sitting pressure and verticality in young adults: A randomized crossover trial

**DOI:** 10.1097/MD.0000000000034625

**Published:** 2023-08-04

**Authors:** Kota Sawa, Keisuke Ishigami, Takuya Miyamoto, Miko Tamura

**Affiliations:** a Department of Physical Therapy, Faculty of Health Sciences, Ryotokuji University, Urayasu, Chiba, Japan; b Department of Rehabilitation, Takenotsuka Noshinkei Rehabilitation Hospital, Adachi-ku, Tokyo, Japan; c Department of Rehabilitation, Tokyo Sakura Hospital, Higashi-Shinozaki, Edogawa-ku, Tokyo, Japan.

**Keywords:** postural balance, sitting position, subjective postural vertical, tilt training

## Abstract

In sensory-motor rehabilitation, sitting-balance training using a wedge may enable optimal postural control. The efficacy and mechanism of achieving lateral adaptation to the sitting pressure and postural vertical are unclear.

**Methods:**

We included 20 healthy participants in a single-blind, randomized crossover trial conducted over 1 day. Sitting pressure was measured while the postural vertical was assessed. The initial training required maximally and laterally inclining the trunk to the left or right, with or without a wedge, 60 times in 2 minutes (0.25 Hz). A repeated-measures 2-way analysis of variance with Bonferroni post hoc analysis was used, and a *P* value < .05 considered statistically significant.

**Results:**

After wedge-adaptation training, the pressure on the weight-bearing surface in the postural vertical position increased only on the left side (*P* < .05). The directional and variability errors of the subjective postural vertical with eyes open and subjective postural vertical were within the range of normative values on verticality.

**Conclusion:**

Wedge-adaptation during sitting training affected sitting pressure and verticality. Therefore, there is potential for future rehabilitation interventions using sitting training with a wedge in individuals with balance disorders.

## 1. Introduction

Postural control is based on interpreting sensory information from the somatosensory, vestibular, and visual systems. The relative sensory reweighting of each of these inputs depends on the goals of the task and environmental context.^[1]^

Although the relationship between balance ability and verticality has been recognized, causality remains unclear. Gravity perception has been discussed as a possible mechanism for verticality judgment, suggesting that it is an important factor in postural control.^[2]^ Sitting pressure is an important cue in assessing gravity because it is possible to evaluate postural control, such as weight-bearing asymmetry (WBA), by evaluating the amount of weight applied from the support surface to the floor in an antigravity posture.^[3]^ WBA is considered to be an evaluation of supportiveness, and it is causally related to postural control. Our results showed that sitting balance training improved variability by enhancing verticality cognition and the ability to perform activities of daily living, as supported by longitudinal studies.^[4,5]^ However, the mechanism by which sitting balance training affects sitting pressure has not yet been clarified. On the other hand, recent reviews have revealed that various sensory and higher brain functions contribute to the vertical perception in balance components. It has been shown that assessing dysfunction related to balance ability is important in rehabilitation interventions and activities of daily living and can be used to predict subsequent outcomes. However, the mechanisms of which are unknown and need further investigation.^[6]^

Therefore, it is important to evaluate the effects and mechanisms of seated balance training using evaluations of center of gravity (CoG) and postural control. We hypothesized that the relationship with gravity perception could be clarified by evaluating the subjective postural vertical (SPV) and analyzing sitting pressure during sitting balance training.

The accurate perception of sitting pressure is essential in maintaining an upright stance; it is a crucial aspect of balance and postural verticality perception by the somatosensory system.^[7]^ Additionally, a perceived deviation in sitting pressure influences the CoG.^[8,9]^ Sitting pressure perception is also affected by gravity in terms of its ability to influence the body postural orientation relative to the earth.^[7,10,11]^ On the other hand, the body somatosensory system influences the perception of sitting pressure.

It has been demonstrated that sitting pressure and SPV were biased toward the left side in a study of the right hemisphere.^[12]^ These authors reported that this phenomenon was caused by right hemisphere dysfunction, which resulted in a leftward bias in sitting pressure and SPV, owing to right hemisphere specificity. In other words, the right hemisphere was mainly responsible for integrating body tilt, somatosensory, and visuospatial cognition. Furthermore, a study conducted on the dominance of the left and right hemispheres found that the right hemisphere controlled the direction and variabilities of the postural vertical more than the left hemisphere.^[13]^ However, it is unclear what type of somatosensory adaptation is present in sitting pressure perception and whether the postural vertical is affected by the sensory input from the body on the left side in healthy participants.

Adapting to sitting pressure as a balance ability is an important aid in stroke rehabilitation because it is predicted to contribute to the reconstruction of sensory integration, balance, and independence. Further, truncal control is an important predictor of recovery.^[14–16]^ Lateral truncal-tilt training can be useful for regaining balance against gravity and the adaptation can be substantial.^[5,17,18]^ The mechanisms of trunk proprioception are affected by the participant training requirements to maintain balance after shifting their weight to a target point. It was suggested that forward reaching of the upper extremity was expected to increase the degree of CoG shift and improve sitting balance ability as its effects.^[19]^ A weight-interventional study demonstrated adaptation in the sitting balance of patients during training^[19]^: specifically, training had an active beneficial effect on truncal function.^[18]^ Several interventional studies of sitting balance have used the tilt board^[19,20]^; however, no previous study has reported using a wedge.

The present study aimed to assess the effect of lateral sitting truncal-tilt training using a wedge to optimize postural control on sitting pressure in healthy adults. The wedge was placed lateral to the body and was expected to influence sitting pressure perception, although the efficacy and the mechanism of the laterality adaptation are not well understood. Therefore, it was hypothesized that the implementation of lateral truncal tilt training using a wedge would enable modification of postural control through changes in sitting pressure and SPV, hence improving balance ability. In addition, this training may be useful for rehabilitation interventions for patients with stroke or Parkinson disease, which present balance disorders.

## 2. Methods

### 2.1. Study design and participants

In this single-blind, randomized crossover trial conducted over the course of 1 day, we recruited 20 healthy volunteers (22.7 ± 1.8 years old) from staff at our hospital between April 2016 and March 2017. We excluded participants with a history of orthopedic pathology or neuromuscular disease.

Written informed consent was obtained from all individual participants included in this study. This clinical trial was registered on the University Hospital Medical Information Network (UMIN)-Center at https://www.umin.ac.jp/ (reference number 000027930) and approved by the Ryotokuji University ethical examination committee (approval number: 00089). Since the University Medical Information Network is considered to meet the International Committee of Medical Journal Editors (ICMJE), an international standard, it was also enrolled in this study.

### 2.2. Randomization

We randomized participants to Control and Experimental groups and also those who received left side measurements. Randomization was concealed from the recruiter and assessors using sealed opaque envelopes containing the allocation, which was generated earlier by a person independent from the study using random number tables, blocked to ensure equal numbers in both groups. The collection of some outcome measures required 2 assessors, who were blinded to group allocation, gave all instructions, and collected the outcome measures post-training.

**Figure 1.**
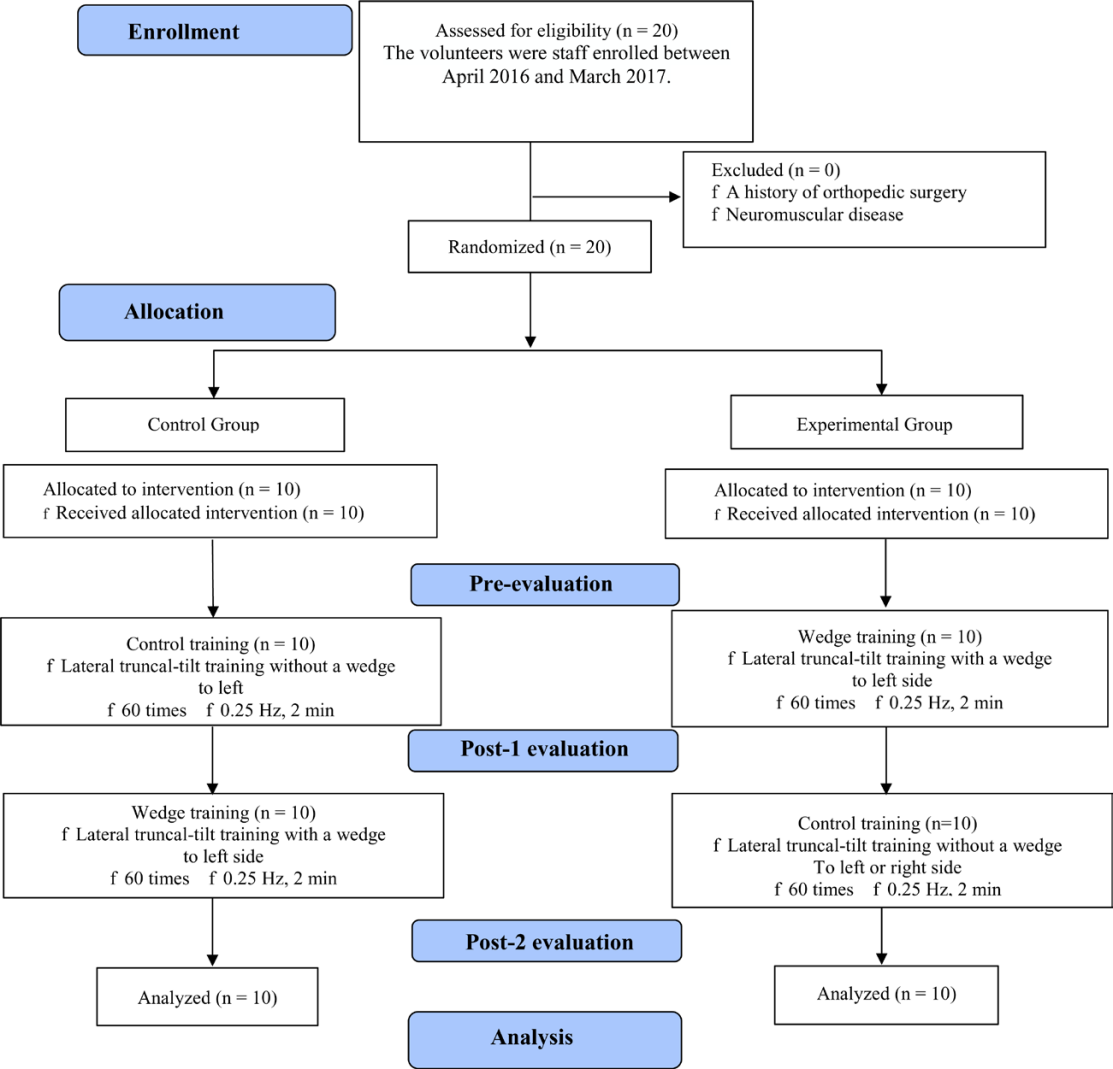
CONSORT flow diagram.

We followed the procedure for selecting and allocating cases advocated by the Consolidated Standards of Reporting Trials (CONSORT) guidelines (Fig. 1).

### 2.3. Apparatus and measurements

**Figure 2.**
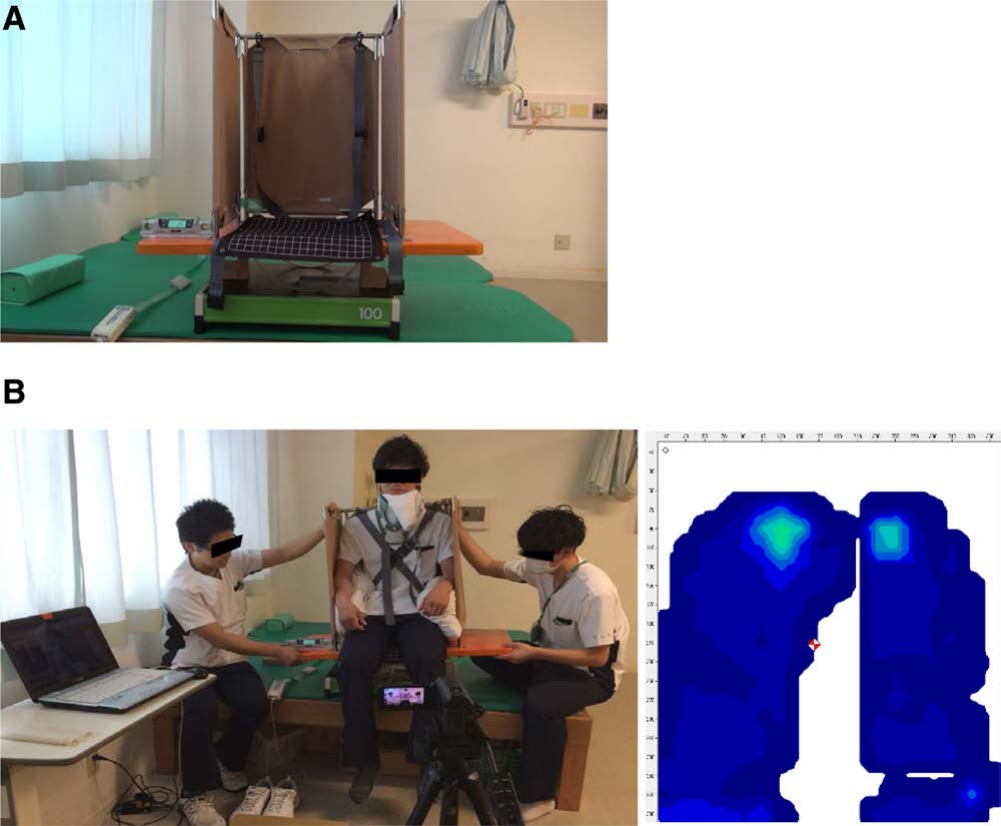
(A) The sitting pressure seat on the postural vertical. (B) Measurement of the postural vertical by means of sitting pressure.

We used sitting pressure measuring equipment (Conform-Light, Nitta Industries Corporation, Tokyo, Japan) to help establish a perpendicular model norm and used the generated pressure measurements to determine what the participants regarded as vertical (Fig. 2A). Sitting pressure (mm Hg) was measured 8 times with eyes open (SPV-Eyes Open; SPV-EO) and SPV judgment. An increase in sitting pressure on the left side was indicated as a “minus” value.

During the SPV-EO and SPV evaluation, we used an originally designed vertical board (VB). The SPV-EO and SPV measurement involved assessing the sitting level on the measuring equipment edge, with (A), a 15° or 20° slant toward the right, followed by (B), a 15° or 20° slant toward the left, moving at a speed of 1.5°/s (Fig. 2B).^[4,21]^ The SPV-EO and SPV measurements were performed on the left and right sides in a counterbalanced order, using ABBABAAB or BAABABBA sequences. Participants indicated when they perceived themselves as being in a true vertical position, and the tilt of the VB was recorded using a digital inclinometer. The slant to the left was denoted as “minus,” and that to the right was denoted as “plus.” The mean values (directional errors) and the standard deviation (variability errors) were calculated across 8 trials for each SPV and SPV-EO test. The measurements were obtained with the patient in a sitting posture wearing a cervical brace to avoid any sense of posture recognition from the neck position (Fig. 2B).

### 2.4. Assessments

We obtained standard medical information for each participants, such as age and sex. Regarding outcomes, the sitting pressure was determined by a physiotherapeutic evaluation at the time of the perpendicular PV assessment. The outcomes were the sitting pressure, SPV-EO, and SPV both pre- and post- the 1 and 2 training protocols (Fig. 1).

### 2.5. Intervention

**Figure 3.**
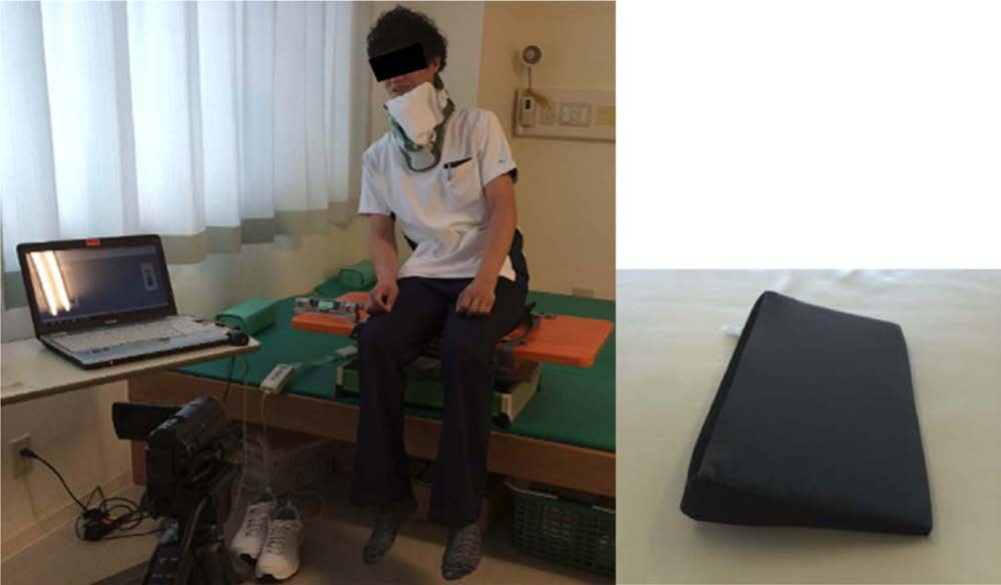
The maximum lateral truncal-tilt training with a 10° wedge.

We used a 10° wedge to produce lateral truncal-tilt training while sitting (Fig. 3).^[4]^ The initial training required maximally and laterally inclining the trunk to the left side, and this movement was repeated 60 times in 2 minutes (Fig. 3) while balancing to the wedge side in the wedge condition in post 1 (Experimental group) and without the wedge in post 2 (Fig. 1). The control condition (Control group) also involved maximally and laterally balancing the trunk to the left side, but without a wedge in post 1 and with the wedge in post 2 (Fig. 1).

Participants’ instructions were as follows: “Tilt your trunk maximally to the left side as far as you can after my signal.” The sitting pressure, SPV-EO, and SPV measurements were performed in a random order on the same day.^[4]^

### 2.6. Statistical analyses

Demographic data were compared between groups using the Mann–Whitney *U* test or chi-squared test. The groups and time-periods were compared in a 2 (with/without wedge) × 3 (at pre, after post 1, and after post 2) analysis with group and time as factors. We used repetition analysis on the pre-training, post 1, and post 2 with or without a wedge in randomized examinations by comparing sitting pressures, SPV-EO, and SPV. The data were analyzed using repeated 2-way analysis of variance.

We examined the main and interaction effects using the Bonferroni method for multiple comparisons. When both effects were present, a simple main effects test was performed using Bonferroni post hoc analysis. SPSS Statistics version 23 (IBM Inc., Armonk, Tokyo, Japan) was used for analyses, and *P* values < .05 were considered statistically significant.

We performed a post hoc power calculation using G*power 3.1 (Heinrich Heine University, Dusseldorf, Germany) to determine the statistical power (1-*β*) and effect size with significance set at 5%. We set the effect size at 0.35 (large effect size) according to *η*^2^ values by Multiple Classification Analysis,^[22]^ 80% power, and between-group differences at pre, left, and right-side evaluations with an α value of 0.05 when employing a crossover study design. Based on this calculated, a sample size of 16 participants per group was required; however, we failed to obtain this sample size due to participant enrollment time-frame restrictions.^[4]^ Moreover, a post hoc power calculation was performed using G*power 3.1 to determine the power (1-*β*) based on the acquired sample size and effect sizes, with significance set at *P <* .05. The effect size for between-group mean differences for the intervention effects was calculated with effect size *f*, and guidelines for interpretation of effect size *η*^2^ were set as follows: 0.02 (small effect size), 0.15 (medium effect size), and 0.35 (large effect size).^[23]^

## 3. Results

### 3.1. Demographic data

Twenty participants (10 each in the control and experimental groups) were included in the study. At baseline, groups did not differ in terms of age, sex, height, and body mass index (Table 1).

**Table 1:** Demographic data.

Items	Control group (n = 10)	Experimental group (n = 10)	*P* value
Age (yr)	23.5 ± 0.8	25.3 ± 2.4	>.05*
Sex	Male 6 Female 4	Male 6 Female 4	>.05†
Right-handed	9	10	>.05†
Height (cm)	161.5 ± 5.6	165.5 ± 7.1	>.05*
Body mass index (kg/m^2^)	19.4 ± 4.7	21.2 ± 6.0	>.05*

* Mann–Whitney *U* test.

† Chi-square test.

### 3.2. Sitting pressures

The pressure on the weight-bearing surface in the SPV-EO was not significantly different at baseline, after post 1, and after post 2 in the experimental training with a wedge group (time, *F* = 1.152, *μ*^2^ = 0.126, *P* = .341; wedge, *F* = 0.520, *μ*^2^ = 0.061, *P* = .491, Table 2). However, the group × time interactions were significant (*F* = 49.276, *μ*^2^ = 0.860, *P* < .001, Table 2). The pressure on the weight-bearing surface in the SPV was not significantly different at baseline, after post 1, and after post 2 in the experimental training with a wedge group (time, *F* = 2.914, *μ*^2^ = 0.267, *P* = .083; wedge, *F* = 0.006, *μ*^2^ = 0.001, *P* = .942, Table 2). However, there was a significant group × time interaction (*F* = 1054.983, *μ*^2^ = 0.992, *P* < .001, Table 2).

**Table 2:** Outcomes of the sitting pressure and postural vertical.

	Verticality		Baseline	Post 1	Post 2	MCID	Interaction F-value	ES (η^2^)	*P value*	Simple main effect F-value	ES (η^2^)	*P value*
Sitting pressure (mm Hg)	SPV-EO	Control	128.5 ± 32.3	141.9 ± 19.8	137.0 ± 22.2	16.15	49.276	0.860	*.000*	T; 1.152	T; 0.126	*T; .341*
		Experiment	136.2 ± 36.7	142.6 ± 22.7†	128.3 ± 19.2	18.35				W; 0.520	W; 0.061	*W; .491*
	SPV	Control	132.2 ± 19.3	146.7 ± 26.6	146.3 ± 10.4	14.65	1054.983	0.992	*.000*	T; 2.914	T; 0.267	*T; .083*
		Experiment	136.5 ± 18.1	148.7 ± 29.8†	136.1 ± 16.4	14.05				W; 0.006	W; 0.001	*W; .942*
Directional errors (°)	SPV-EO	Control	0.5 ± 0.7	0.4 ± 0.7	1.4 ± 1.1	0.35	76.968	0.895	*.000*	T; 0.280	T; 0.065	*T; .763*
		Experiment	1.0 ± 0.7	0.6 ± 1.0†	0.9 ± 0.7	0.35				W; 3.012	W; 0.251	*W; 0.117*
	SPV	Control	0.6 ± 0.9	0.1 ± 1.1	1.4 ± 1.3	0.45	16.358	0.645	*.003*	T; 0.654	T; 0.068	*T; 0.532*
		Experiment	0.3 ± 1.5	0.8 ± 1.5	0.1 ± 1.0†	0.75				W; 0.028	W; 0.003	*W; 0.872*
Variability errors (°)	SPV-EO	Control	2.7 ± 0.9	1.9 ± 0.8*	2.0 ± 1.0	0.45	254.246	0.966	*.000*	T; 14.105	T; 0.610	*T; .000*
		Experiment	2.8 ± 1.5	2.0 ± 0.8†,*	2.3 ± 1.1	0.75				W; 0.122	W; 0.013	*W; .735*
	SPV	Control	3.1 ± 1.3	2.3 ± 0.9*	2.6 ± 0.7	0.65	165.047	0.948	*.000*	T; 6.853	T; 0.432	*T; .006*
		Experiment	2.8 ± 1.6	1.9 ± 0.8†,*	2.3 ± 0.9	0.80				W; 0.083	W; 0.009	*W; .780*

ES = effect size; MCID = minimal clinically important difference; SPV = subjective postural vertical; SPV-EO = SPV-eyes open; T = time; W = wedge.

*The simple main effects of time (T) or wedge (W) (*P* < .05).

† The interactions of time and group (*P* < 0.05).

### 3.3. Subjective postural vertical with eyes-opened and subjective postural vertical

The directional errors of SPV-EO were not significantly different at baseline, after post 1, and after post 2 in the experimental training with a wedge group (time, *F* = 0.280, *μ*^2^ = 0.065, *P* = .763; wedge, *F* = 3.012, *μ*^2^ = 0.251, *P* = .117, Table 2). However, there was a significant interaction between time-point and group (*F* = 76. 968, *μ*^2^ = 0.895, *P* < .001, Table 2). SPV-EO variability was significantly different between baseline, after post 1, and after post 2 in the experimental training with a wedge group (time, *F* = 14.105 *μ*^2^ = 0.610, *P* < .001; wedge, *F* = 0.122, *μ*^2^ = 0.013, *P* = .735, Table 2). There was also a significant group × time interaction (*F* = 254.246, *μ*^2^ = 0.966, *P* < .001, Table 2).

The directional errors of SPV were not significantly different at baseline, after post 1, and after post 2 in the experimental training with a wedge group (time, *F* = 0.654, *μ*^2^ = 0.068, *P* = .532; wedge, *F* = 0.028, *μ*^2^ = 0.003, *P* = .872, Table 2). However, there was a significant interaction between time-point and group (*F* = 16.358, *μ*^2^ = 0.645, *P* = .003, Table 2). The variabilities of SPV were significantly different between baseline, after post 1, and after post 2 in the experimental training with a wedge group in terms of simple main effects (time, *F* = 6.853, *μ*^2^ = 0.432, *P* = .006; Wedge, *F* = 0.083, *μ*^2^ = 0.009, *P* = .780, Table 2). Moreover, the interaction between time-point and group was significant (*F* = 165.047, *μ*^2^ = 0.948, *P* < .001, Table 2).

## 4. Discussion

### 4.1. Sitting pressure

The sitting pressure at the time of the SPV judgment showed that postural change was based on visual, somatosensory, and gravity perception. Sitting pressure during the SPV judgment was notably different between the control and experimental conditions and between the left- and right-side tilts. The wedge balance training with maximum lateral truncal tilt changed the directional errors on the left side, and variability errors in SPV judgment occurred. The balance training significantly affected the weight-bearing sitting pressure distribution during the SPV judgment. The relationship between WBA in postural balance and sitting pressure, caused by sensory adaptation in body tilt, and balance strategies have been previously reported.^[3]^ In the present study, the postural adaptation of balance differed from the visual adaptation; therefore, the body tilt adaptation with a wedge used somatosensory orientation for postural control, and the visual adaptation was cognitive. The wedge adaptation specifically affected the SPV variability errors on the left side of the body. This suggests that stimulation of spinal and cortical vestibular tracts and somatosensory information stimulated the righting reactions, which enabled the CoG to shift with the weighting and cognitive load in the eyes-closed postural orientation. As most people are right-handed, their leading foot is usually on the left side in terms of laterality in human higher-brain function. This means that wedge insertion on the left side may effectively correct posture in terms of the body axis. Therefore, it is likely that further adaptation will occur if the left side is corrected first in the sitting lateral-tilt training task. Our results suggest that using sitting pressure had a positive effect on eliciting trunk alignment and adjustment of the postural setting in the eyes-closed postural control training task.

### 4.2. Subjective postural vertical with eyes-opened and subjective postural vertical

The directional errors of SPV-EO showed no significant main effects of time nor group (wedge condition) in the control and experimental groups. However, the interaction of time and group showed a significant difference between the control and experimental conditions. Nevertheless, our results were obtained in healthy individuals, in whom stability was not significantly higher on the left side than on the right side (only minimal clinically important differences). SPV directional errors were within the normal range (0.12° ± 0.95° in the roll plane and −2.5° ± 2.5° in the roll plane).^[12,13]^ Thus, there was no error of bias regarding vertical perception. In addition, it has been reported that directional tilt errors of SPV were 0.1° ± 0.6° in young, healthy participants and −0.1° ± 1.1° in older participants.^[24]^ The underlying mechanisms involved sensory-motor adaptations in the vertical plane, and a dynamic balance strategy was used for errorless control of the postural vertical in humans.^[25]^

In the present study, the directional errors of SPV showed no significant differences in terms of simple main effects after training in the experimental condition. The variability of errors of SPV was significantly different in terms of time and group interactions. Therefore, somatosensory wedge adaptation was symmetrical in the vertical axis. However, adaptation did not occur only on the left side in our healthy participants, as values were within the minimal clinically important differences.

### 4.3. Limitations

This study has some limitations. The study had a small sample size; therefore, further studies examining the long-term effects of balance training are necessary to confirm the results found in the current investigation. Moreover, a possibility of bias existed owing to the survey evaluation methods.

## 5. Conclusion

This study examined the impact of sensory-motor wedge adaptation on sitting balance training using a wedge, which has not been reported in previous studies. Optimal postural control may be achieved by increasing the sitting pressure on the weight-bearing surface in the SPV by training. The directional and variability errors of SPV-EO and SPV were in the normative range for verticality. Nevertheless, the maximum lateral truncal-tilt balance training using a wedge resulted in improved sitting pressure distribution during SPV judgment. Accordingly, there is potential for future rehabilitation interventions involving sitting balance training using a wedge in cases of balance disorders.

## Acknowledgments

The author thanks hospital staff for their assistance in analyzing the number of samples of healthy participants.

## Author contributions

**Data curation:** Kota Sawa, Keisuke Ishigami, Takuya Miyamoto, Miko Tamura.

**Formal analysis:** Kota Sawa.

**Project administration:** Kota Sawa.

**Writing – original draft:** Kota Sawa.

**Writing – review & editing:** Kota Sawa.
